# Early verrucous cell carcinoma of the esophagus: a case report and endoscopic and histologic features

**DOI:** 10.1186/s12876-021-02049-0

**Published:** 2021-12-14

**Authors:** Lianjun Di, Xinglong Wu, Zhenyu Chen, Jianhong Zhu, Haibo Wang, Biguang Tuo

**Affiliations:** 1 Department of Gastroenterology, Affiliated Hospital of Zunyi Medical University, Zunyi, 563003 China; 2 Department of Pathology, Affiliated Hospital of Zunyi Medical University, Zunyi, China; 3 Department of Gastroenterology, Nan Fang Hospital, Southern Medical University, Guangzhou, China; 4 Department of Gastroenterology, The Second Affiliated Hospital of Soochow University, Suzhou, China

**Keywords:** Case report, Early diagnosis, Endoscopic feature, Esophagus, Verrucous cell carcinoma

## Abstract

**Background:**

Verrucous cell carcinoma of the esophagus (VCCE) is an extremely rare tumor and generally detected at advanced stage. Despite of its slow growth and well differentiation, it has very poor prognosis with high mortality. Therefore, early detection is a critical to improve patients’ survival. However, no early cases of VCCE have been reported and the endoscopic features of early VCCE are not well described. We herein report the endoscopic and histologic features of an early VCCE.

**Case presentation:**

A 54-year-old man with a history of excessive alcohol and tobacco use was admitted to our hospital because of chronic persistent swallowing dysfunction for six months. White light endoscopy revealed a flat lesion covered with scattered leukoplakia in the middle esophagus. Magnifying endoscopy with narrow-band imaging showed tiny irregular papillary microsurface structure. The lesion was considered as early esophageal cancer and completely resected with endoscopic submucosal dissection. Histological examination confirmed that the lesion was early VCCE which was limited within the mucosal lamina propria (m2).

**Conclusion:**

VCCE is rare with poor prognosis. This is a report of early VCCE and description of its endoscopic features which will contribute to early detection of these cancers.

## Background

Verrucous cell carcinoma is a rare variant of squamous cell carcinoma, which is characterized as a slow growing, well-differentiated, and locally invading tumour. It is usually seen in oropharynx, larynx, and genitalia. Verrucous cell carcinoma of the esophagus (VCCE) is extremely rare [1, 2]. The first case of VCCE was reported by Minielly et al. [3] and less than 50 cases have been reported worldwide so far [4]. The clinical presentation of VCCE currently lacks specificity. It is mainly detected by endoscopy. The diagnosis of VCCE is very difficult because the superficial layer of the tumour is covered by non-malignant tissue and it is generally detected at advanced stage [5]. Despite its slow growth and well differentiation, it has very poor prognosis with high mortality because of the delay between the initial presentation and the diagnosis [6]. Therefore, early detection is a critical to improve the prognosis of VCCE. However, early VCCE is rarely found. There was no report of early VCCE and the description of its endoscopic and histopathological features. We herein report the endoscopic and histopathological features of an early VCCE limited within the mucosal lamina propria (M2) which may contribute to the early detection of VCCE.

## Case presentation

**Fig. 1.**
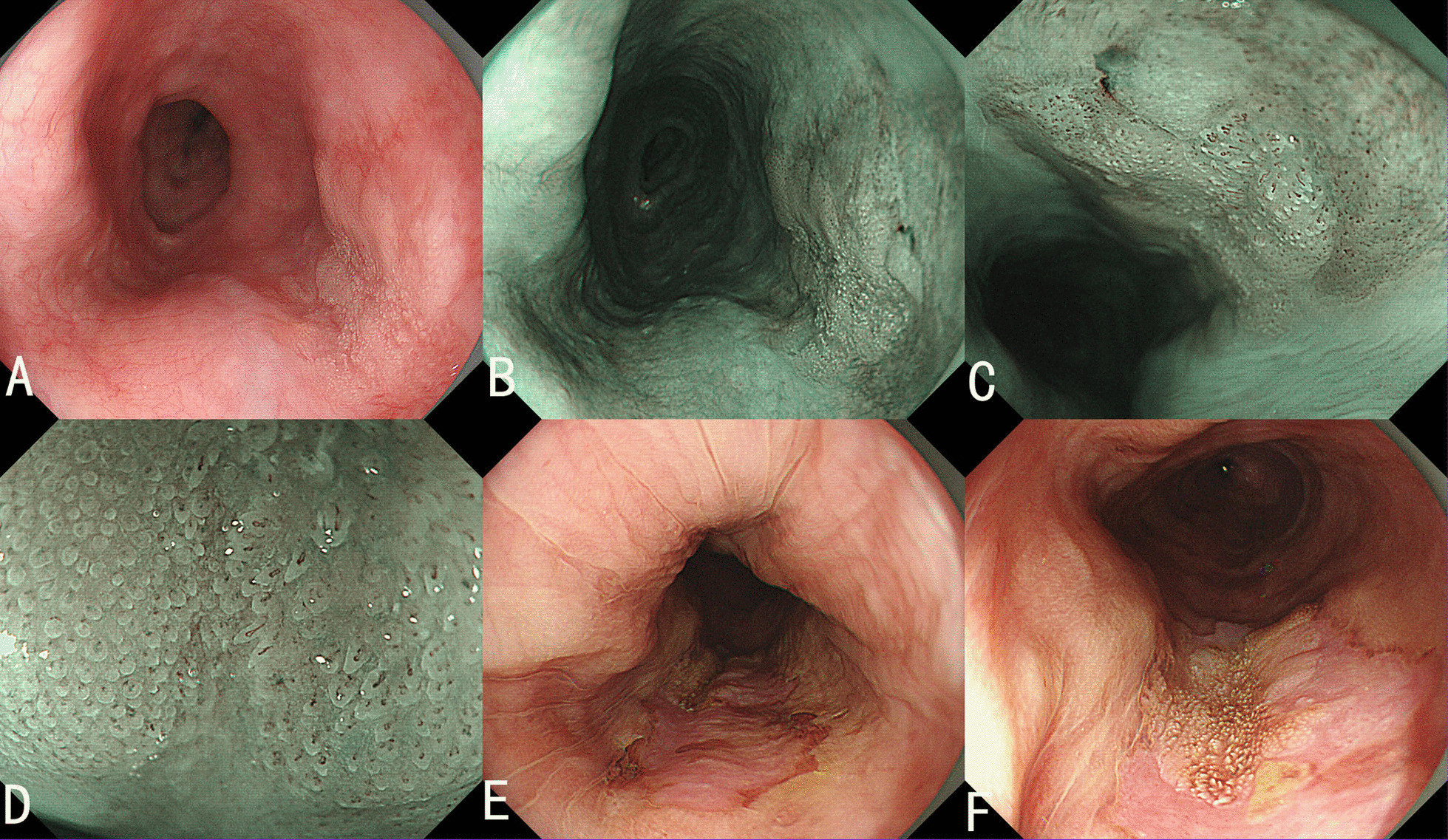
Endoscopic manifestation. **a** WLE shows a lesion with scattered leukoplakia and absence of normal vascular network. **b** NBI shows the lesion with an indistinct brownish area, local white penniform area, and white tiny papillary surface structure. ME-NBI (**c**, low ME-NBI; **d**, high ME-NBI) shows tiny irregular papillary microsurface structure with various shapes and sizes, and non-typical type B1 pattern of IPCLs confined to the papillary microsurface structure. Iodine staining endoscopy shows a less-stained lesion (**e**) and more distinct white tiny papillary surface structure (**f**).

A 54-year-old man with a history of excessive intake of alcohol (100 g/day × 30 years) and locally grown tobacco use (500g/month × 30 years) was admitted to our hospital because of chronic persistent swallowing dysfunction for six months. White light endoscopy (WLE) revealed a 22 mm flat lesion in the middle esophagus.The lesion was covered with scattered leukoplakia, and normal vascular network could not be seen in the lesion (Fig. 1a). Narrow-band imaging (NBI) under endoscopy revealed the lesion with an indistinct brownish area, local white penniform area, and white tiny papillary surface structure (Fig. 1b). Further low and high magnifying endoscopy with NBI (ME-NBI) showed tiny irregular papillary microsurface structure with various shapes and sizes, and non-typical type B1 pattern of intraepithelial papillary capillary loops (IPCL) confined to the papillary microsurface structure (Fig. 1c, low magnification, and Fig. 1d, high magnification) according to the classification of Japan Esophagus Society (JES classification) [7]. Iodine staining endoscopy revealed a less-stained lesion (Fig. 1e) and showed more distinct white tiny papillary surface structure (Fig. 1f) than conventional WLE. Biopsy showed a low grade intraepithelial neoplasia. Chest and abdominal CT scan revealed no abnormal lesions.

**Fig. 2.**
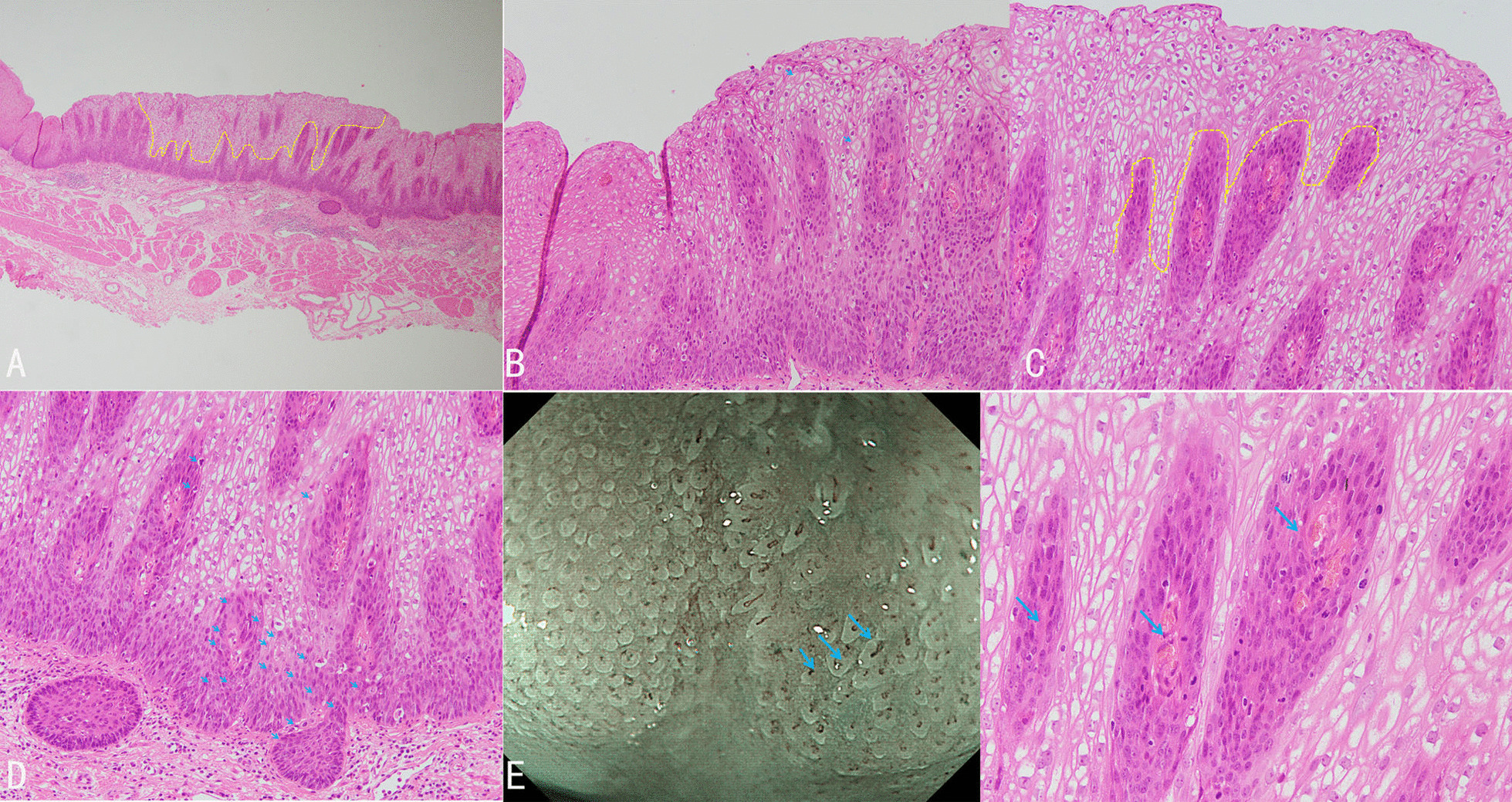
Histologic manifestation. **a** Thickened neoplastic spinous cell layer. **b** Local keratosis presenting the appearance of church spire. **c** Elongated lamina propria papilla upward to the surface layer. **d** Tumor cells with double or more nucleoli in the basal region, and tumor cells breaks through the basement barrier and invades superficial lamina propria. **e** IPCLs observed by microscopy (right) and endoscopy (left).

**Fig. 3.**
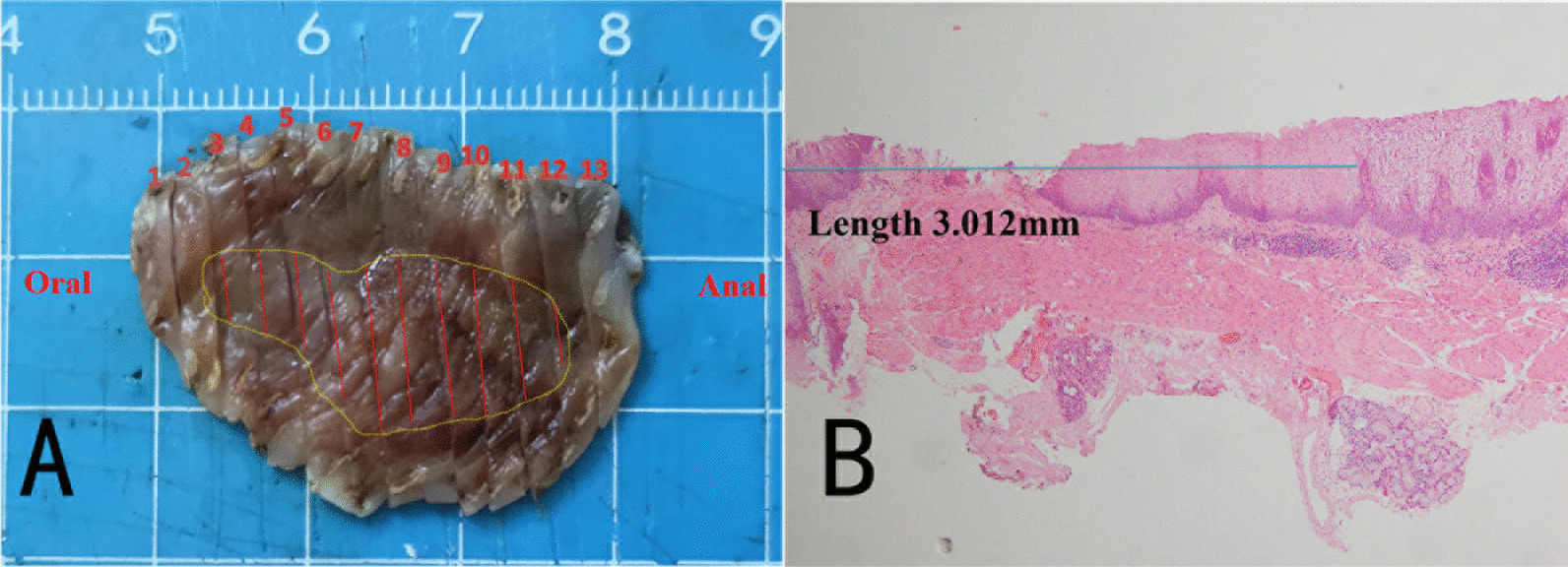
Contrastive analysis for the resected specimen and histopathologic examination. **a** The resected specimen was cut into slices at each 2.5 mm width. The red lines represent lesion areas in each slice. Oral is oral margin of the specimen. Anal is anal margin of the specimen. **b** Histopathology showing the distance of the lesion to the closest margin of the resected specimen

According to the endoscopic manifestations under WLE, ME-NBI, and iodine staining endoscopy, combining with pathology, we considered the lesion to be early esophageal cancer. Therefore we chose endoscopic therapy. The lesion was completely resected with endoscopic submucosal dissection. Histopathology showed that the layer of neoplastic spinous cells was significantly thickened (Fig. 2a) with local keratosis presenting the appearance of the so-called church spire (Fig. 2b). The lamina propria papilla was elongated upward to the surface layer (Fig. 2c), and the nuclei in the basal region had double or more nucleoli. Tumor cell displayed expanding growth, and partial tumor cells broke through the basement barrier and invaded into superficial lamina propria (Fig. 2d). The IPCL were observed in lamina propria papilla with elongating upward to the surface layer growing underneath the layer of neoplastic spinous cells, which corresponds to the IPCL observed by ME-NBI (Fig. 2e). The lesion was histopathologically confirmed as early VCCE which was limited within the mucosal lamina propria (m2) and completely resected. There were no lymphovascular and neural invasions of cancer cells, and no cancer cells to be seen in the lateral and vertical margin of the resected specimen (the distance of the lesion to the closest margin of the resected specimen was 3.012mm) (Fig. 3).

## Discussion and conclusions

VCCE is a very rare histological variant of squamous cell carcinoma and so far less than 50 cases are reported in the world [4]. The etiology of VCCE is not clear and it seems to be associated with chronic inflammatory conditions or a long term local disease process. The common risk factors are smoking and alcohol abuse [6]. In this case, the patient had a long term and heavy medical history of smoking and alcohol abuse. The most common chief complaint was dysphagia, seen in our case as well. Other symptoms would be hematemesis, coughing, and odynophagia [6, 8]. VCCE is detected mainly by endoscopy. It is very difficult to diagnose and often delayed since initial superficial biopsies usually show nonspecific findings of acanthosis, hyperkeratosis, and parakeratosis with associated acute and chronic inflammation, with no sign of malignancy.

The described endoscopic appearance of VCCE includes a shaggy, velvety, or papillary mucosa with white plaques and exophytic, wartlike, or cauliflower-like mass. In this case, WLE revealed a flat lesion in the middle esophagus which was covered with scattered leukoplakia. The lesion displayed an indistinct brownish area, local white penniform area, and white tiny papillary surface structure under NBI endoscopy. ME-NBI showed tiny irregular papillary microsurface structure with various shapes and sizes, and a non-typical type B1 pattern of IPCL confined to the papillary microsurface structure. Histologically, the IPCLs were observed in lamina propria papilla with elongating upward to the surface layer growing underneath the layer of neoplastic spinous cells. In addition, histology also revealed that the tumor cell displayed expanding growth, and partial tumor cells broke through the basement barrier and invaded superficial lamina propria, indicating that the lesion tends to be deep-growing and invasive.

VECC is a slowly growing and well-differentiated variant of squamous cell carcinoma, and none of the reported cases showed distant metastases [9], but it has very poor prognosis because there is a delay between the initial presentation and the diagnosis, and majority of the cases are detected at advanced stage. Morbidity and mortality of the disease are mainly due to local invasion or surgical complications. Therefore, early detection is critical to improve patient’s survival. In this case, it demonstrates that NBI and ME-NBI under endoscopy contribute the early detection of VECC. High index of suspicion is required based on endoscopic appearance. In this case, biopsy from the lesion showed a low grade intraepithelial neoplasia, but according to the endoscopic manifestations under WLE, ME-NBI, and iodine staining endoscopy, we considered the lesion to be early esophageal cancer and chose endoscopic resection, and the lesion was confirmed as VECC.

In conclusion, VECC is a rare variant of squamous cell carcinoma with a poor prognosis. Early detection is critical to improve patient’s survival. We herein present the endoscopic and histological features of an early VECC. To our knowledge, this is the report of the earliest VECC and the description of its endoscopic features to date, which contributes to promote the early detection of VECC.

## Data Availability

All data and material generated or analysed during this study are included in this published article.

## Abbreviations

IPCL: Intraepithelial papillary capillary loops
ME-NBI: Magnifying endoscopy with narrow-band imaging
NBI: Narrow-band imaging
VCCE: Verrucous cell carcinoma of the esophagus
WLE: White light endoscopy
